# Brain functional training: a perspective article

**DOI:** 10.3389/fragi.2024.1368878

**Published:** 2024-06-21

**Authors:** Marzo Edir Da Silva-Grigoletto, Marcos Raphael Pereira-Monteiro, José Carlos Aragão-Santos, Alan Bruno Silva Vasconcelos, Pablo Jorge Marcos-Pardo, Leonardo de Sousa Fortes

**Affiliations:** ^1^ Department of Physical Education, Federal University of Sergipe, São Cristóvão, Sergipe, Brazil; ^2^ Graduate Program in Physiological Sciences (PROCFIS), Federal University of Sergipe, São Cristóvão, Sergipe, Brazil; ^3^ Graduate Program in Health Sciences (PPGCS), Federal University of Sergipe, Aracaju, Sergipe, Brazil; ^4^ Department of Health Education, Federal Univesity of Sergipe, Lagarto, Sergipe, Brazil; ^5^ Department of Education, Faculty of Educational Sciences, University of Almería, Almería, Spain; ^6^ Associate Graduate Program of Physical Education, Federal University of Paraíba, João Pessoa, Brazil

**Keywords:** exercise, cognitive training, functional status, aging, health

## Abstract

**Introduction:** Physical exercise (PE) positively affects the nervous system, impacting morphology and physiology. It increases brain gray and white matter, improves cerebral blood flow, and stimulates neurogenesis, synaptogenesis, and angiogenesis, promoting brain function. Although exercise already affects cognition, some training modalities place greater demands on the cognitive aspects of physical exercise, such as perceptual-motor and visual-motor training. This type of approach aims to emphasize the cognitive adaptations that occur chronically. Specifically for older people, functional training, a multi-component approach, is a promising exercise modality that stimulates functionality using multi-joint, multi-planar exercises mirroring daily activities. However, applying a greater focus on cognitive adaptations in line with the functional training proposal for maximal benefits remains underexplored.

**Aim:** Thus, this perspective article initially explores different exercise approaches emphasizing cognitive adaptations and proposes Brain Functional Training to improve older adult’s functionality.

**Methods:** Furthermore, we explain how brain functional training can be explored to emphasize cognitive aspects based on increasing complexity to stimulate the executive function and its subdomains.

**Conclusion:** This proposal is one alternative to combining motor and cognitive stimuli to promote autonomy and health in older people.

## 1 Introduction

Physical exercise programs (PEP) has numerous beneficial effects on human morphology, and central and peripheral nervous system functions due to physiological processes in response to physical stress (Mandolesi et al., 2018; Mahalakshmi et al., 2020). In a chronic way, PEP can increase the volume of gray and white matter in the brain, promote neurogenesis, synaptogenesis, and angiogenesis, and stimulate brain plasticity (Kokubun et al., 2021; Cuyul-Vásquez et al., 2023). Among the possible ways in which PEP can affect cognitive function are structural changes in the hippocampus, promotion of mitochondrial health, cytokine secretion, promotion of brain metabolism, and regulation of gut microflora (Lu et al., 2023).

PEP significantly affects the brain subcortical and peripheral areas of the nervous system that play essential roles in motor control, regulation of the autonomic system, and various functions related to health and wellbeing (Morgan et al., 2015). Specifically, PEP influences the functions of the basal ganglia (Zikereya et al., 2023) and cerebellum (Jalanko et al., 2023; Purcell et al., 2023), improving motor coordination, precision, balance, and posture. Also, PEP helps regulate the body’s autonomous functions, such as controlling heart and respiratory rates, blood pressure, and digestion (Monda et al., 2017; Nystoriak and Bhatnagar, 2018). All these effects of PEP are translated into improved health and quality of life. It is important to note that the load, volume, intensity and modality presented are important factors in modulating the effects of PEP.

Different training modalities, such as perceptual-motor, visual-motor, and ideo-motor training, have been proposed to stimulate neural and cognitive aspects. In the last decade, functional training, defined as a multicomponent approach of training that emphasizes the functionality utilizing multiarticular motor patterns exercises, has been highlighted due to its transferability to activities of daily living and the use of complexity as a progression strategy (Da Silva-Grigoletto et al., 2014; Stenger, 2018). In addition to the effects of physical exercise on the nervous system mediated by indirect factors, we hypothesize that increased complexity will promote increased connectivity between the different cortical regions leading to even more benefits of the training program on functionality. Then, this perspective article aimed to present and briefly discuss the increasing complexity in the tasks performed during the functional training session in order to improve cognitive increases, proposing a novel training approach called Brain-Functional Training.

## 2 Motor-cognitive approaches

The scientific literature has associated PEP with cognitive tasks to promote functionality in individuals with impaired cognition (Thom and Clare, 2011). Practical proposals were presented integrating physical exercise and cognitive tasks based on principles of cognitive psychology. This type of intervention is called psycho-cognitive motor training (Pittera, 2017). Training modalities using motor and cognitive tasks simultaneously are generically called cognitive-motor training and theoretically promote higher brain activity. According to Di Santo^1^, this type of training aims to improve cortex activation, in an acute way, and intensify cognitive adaptations chronically. These phenomena are found in some recent studies in the scientific literature (Díaz-García et al., 2023; Staiano et al., 2023; Béraud-Peigné et al., 2024; Lee et al., 2024).

In the intention of developing perceptual skills, which is very important in sports (Farrow, 2013), perceptual-motor training is commonly used, combining exercises that challenge gross and fine motor skills, such as coordination and balance, with perceptual tasks (Soltani Kouhbanani et al., 2020), affecting executive functions and decision-making responses (Farrow, 2013; Milazzo et al., 2016; Soltani Kouhbanani et al., 2020; Li et al., 2023). The physiological mechanisms underlying the motor ability are related to the connectivity of the brain areas (Hung et al., 2013). By integrating different cognitive-motor tasks, perceptual-motor training includes visual localization tasks (Milazzo et al., 2016; Fooken et al., 2018). Including visual tasks gave rise to visuomotor training, one different approach that modulates neural factors such as corticomotor reorganization and neural plasticity (Hülsdünker et al., 2018; Canegallo, 2019; Cavaleri et al., 2020).

There are, basically, two possibilities for using the dual-task exercises. The first way is based on adding motor or cognitive tasks in different moments sequentially or simultaneously implementing both stimuli (Herold et al., 2018). Specifically, emphasizing the functionality principle, the simultaneous approach is more similar to activities of daily living (Tait et al., 2017). Furthermore, the simultaneous approach allows the use of additional or incorporated tasks. The additional tasks approach combines a motor and a cognitive task that are performed simultaneously but are independent of the other (e.g., walking while performing an arithmetic operation). In contrast, the incorporated tasks approach implies that the cognitive tasks must be completed correctly to accomplish the motor task (e.g., walking in a specific path resulting from a mathematical operation performed while walking).

It is essential to highlight that the perception of these different demands (physical and cognitive) can increase the participant’s motivation and the adherence of the public (Mahmood et al., 2023). Specifically, about adherence, the strategies used to challenge the planning (increased complexity and periodic changes of stimuli) tend to affect the self-efficacy and consequently improve adherence (Collado-Mateo et al., 2021). In the same way, we can perform a cognitive dual-task, such as walking while counting numbers, we can choose the motor dual-task, such as walking while holding one ball (Liu et al., 2017; Pantoja-Cardoso et al., 2023). This way can increase the complexity of the exercise and impact cognitive adaptations (Sudo et al., 2022).

## 3 Brain functional training

Functional training is a multi-component approach emphasizing the synergistic, integrated, and balanced improvement of various physical capacities through multi-joint and multi-planar exercises that mimic patterns observed in daily activities. Based on the specificity principle, this training approach aims to ensure efficiency and safety during daily activities (La Scala Teixeira et al., 2017; Da Silva-Grigoletto et al., 2020). Moreover, functional training uses complexity as a form of progression, aligning exercises with everyday situations aligned with current guidelines highlighting the importance of human functionality, especially for older people (Fragala et al., 2019; Izquierdo et al., 2021).

To amplify the effects of functional training, we understand the need to incorporate tasks emphasizing perceptual-cognitive aspects into training sessions. This inclusion addresses the inherent complexity of daily actions’ physical and cognitive demands. This need arises when we notice that changes in the volume and intensity of traditional physical exercise models show increases in the excitability of the corticospinal pathway but do not present differences in the intracortical pathways (Flanagan et al., 2012; Latella et al., 2017). In summary, the connectivity between the different cortical regions is not increased during traditional training, only the connectivity between the cortical regions with the locomotor system. In fact, it is currently seen that as the intensity-load increases there is deactivation of some cortical areas, mainly related to cognition, while the motor areas remain activated (Fontes et al., 2020).

Therefore, we propose a training approach that includes a greater demand for intracortical connections, generated by acute training sessions, providing chronic neural adaptations related to greater performance in the planning. It is worth pointing out that planning is an important executive function resulting from the interaction between inhibitory control, cognitive flexibility, and working memory (Diamond, 2013). The hypothesis for this improvement stems from previous studies that found improvements in executive functions and physical performance through the repeated application of protocols that combine the use of physical and cognitive tasks (Dallaway et al., 2021; Díaz-García et al., 2023; Lima-Junior et al., 2023; Staiano et al., 2023). In addition, a previous study by Dallaway et al. (2023) showed that performing a mental task before a muscle endurance training protocol could increase oxygenation in the prefrontal region and endurance performance. In this sense, we believe that tasks linked to greater demand on the core executive functions are aligned with the individual’s functionality.

Scientific literature presents nomenclature proposals using “Brain” combined with the modality, such as Concurrent Brain Endurance Training or Brain Endurance Training (Dallaway et al., 2021; Lima-Junior et al., 2023). In this regard, we propose “Brain Functional Training” as it allows motor focus on daily tasks while increasing the demand for higher cognitive processes. This approach advocates for simultaneous training of physical and cognitive aspects in a single training session, potentially being a suitable training option for populations with physical and cognitive deficits, such as older people. Furthermore, in Brain Functional Training, it would be possible to individualize the external load of physical training and the cognitive training load, considering the quantity and complexity of physical exercises, as well as the quantity and complexity of cognitive stimuli.

**FIGURE 1:**
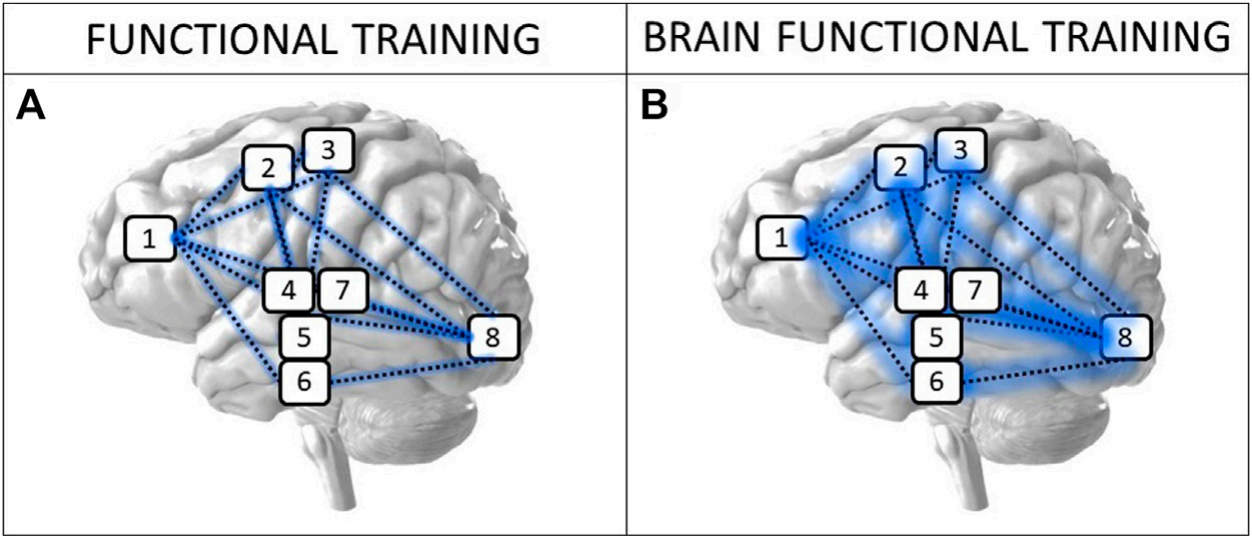
Main brain areas that undergo increased activation when modified from the classic FunctionalTraining approach **(A)** to the BFT approach **(B)**. Note:1: Prefrontal cortex (PFC); 2: Supplementary Motor Area (SMA); 3: Primary Motor Cortex (M1); 4: Basal Nuclei; 5: Lateral Genicular Nucleus (GLN); 6: Midtemporal Cortex (MTC); 7: Thalamus; and 8: Visual Cortex (VC).

The choice of exercises for this training approach involves a balance between selecting the motor complexity for each task (e.g., in older people: Lying/sitting → Standing; Uni-segmental → Multi-segmental; Uni-planar or one-dimensional → Multi-planar or three-dimensional; Slow → Fast; Cyclic → Acyclic; Bilateral → Unilateral; Motor single task → Motor dual task) and associating them with cognitive tasks (e.g., Stroop Task; Go/No Go Task; N-Back Task) aiming to improve the connection of higher brain centers, for instance, the prefrontal cortex, motor areas, basal nuclei, and visual cortex. Figure 1 shows a hypothetical, schematic, and illustrative model in which we can see the increased connectivity between the main brain areas involved during Functional Training (A) and BFT (B). This hypothetical model should be clarified in further studies. It is worth pointing out that attentional cost in such tasks is high at the beginning of the learning process, but reduces with practice. In this sense, to maintain motor and cognitive stimuli, it is necessary to periodically increase the complexity of the tasks, preferably every a few weeks (Staiano et al., 2023).

Additionally, we understand the tasks should be performed simultaneously, characterized as a dual-task configuration, as this resembles daily tasks. For example, it is possible to use a pulling pattern associated with an N-back task, aiming for improvements in strength and working memory with a single exercise. Another example is performing a horizontal med ball throw associated with an inhibition of a target selection upon the instructor’s command, aiming to improve muscular power and inhibitory control function.

Brain Functional Training offers various exercise choices and combinations with different dual-task strategies. These combinations can, in the long run (chronically), increase connectivity of brain areas involved in the reward system (e.g., basal nuclei and PFC). Thus, Brain Functional Training emerges as a prominent approach, considering motivational aspects and, consequently, adherence to the training program (Collado-Mateo et al., 2021).

## 4 Discussion

This innovative proposal emphasizes the need to integrate different physical capacities and the simultaneous stimulation of cognitive processes. This makes Functional Training, which promotes various benefits for the older population, even more similar to daily activities, which constantly require the management of combined tasks from both a motor and cognitive perspective. This need for interaction between thinking and performing tasks is greater at the start of training, or in new sessions, due to the learning process. In this sense, new stimuli in complexity are needed periodically to improve this motor and cognitive interaction. Thus, the present proposal has high applicability, considering the promotion of autonomy and independence for older people.

In using Brain Functional Training, it is essential to quantify the external load of the total stimulus applied, as in any other training approach. However, since cognitive load plays a determining role in motor performance, the ability to discriminate how much the motor and cognitive stimuli affect the individual’s performance becomes necessary. Therefore, we recommend using the perceived exertion scale for quantifying the total effort, as this method is recommended for quantifying load in multi-component modalities (Falk Neto et al., 2020), such as functional training. Furthermore, we suggest using metrics to assess the cognitive demands required, which can be done more accurately through imaging tests or psychophysiological tests. However, it is worth pointing out that the use of imaging tests during the performance of Functional Training protocols is unfeasible because most exercises are multiplanar and multi-joint. In this sense, some possibilities are the use of the percentage delta of psychometric tasks between before and after protocol or the use of the NASA Task Load Index (Riley et al., 2021). The literature also includes the use of equipment with good discrimination capacity for mental load indicator variables, such as the eye tracker (Lima-Junior et al., 2023). When it is impossible to carry out more accurate tests, we recommend looking for ecological strategies such as the cognitive load scale (Ouwehand et al., 2021), understanding that validation and transcultural adaptation processes are necessary for the use of scales.

We suggest that the scientific researchers interested in the field conducts studies that explore the mechanisms involved in the physiological effects promoted by BFT on executive function and functionality, including possible intervention proposals. We also believe there is a need for clinical trials comparing BFT with other modalities commonly used in the literature with regard to its chronic effects on aspects of functionality and executive function. This approach has great potential for health promotion and closely resembles daily life activities by introducing complexity to exercise and requiring cognitive processes. Therefore, several questions about the ideal balance between cognitive and motor loads in different clinical contexts must be elucidated.

## Data Availability

The original contributions presented in the study are included in the article/Supplementary material, further inquiries can be directed to the corresponding author.

## Footnotes

1 Di Santo, M. Didactic material provided by the course “Professional Diploma in Neurobiology and Psychology in Team Sports” (Barça Innovation hub).
